# Identity Theft and Older Adults: How Minorities and the Poor Suffer the Worst Consequences

**DOI:** 10.1093/geroni/igab046.1256

**Published:** 2021-12-17

**Authors:** Marguerite DeLiema, David Burnes, Lynn Langton

**Affiliations:** 1 University of Minnesota, Twin Cities, SAINT PAUL, Minnesota, United States; 2 University of Toronto, Toronto, Ontario, Canada; 3 RTI International, RTI International, District of Columbia, United States

## Abstract

Society’s growing reliance on technology to transfer and store private information has created more opportunities for identity thieves to access personal data. Prior work using data from the National Crime Victimization Survey (NCVS) Identity Theft Supplement (ITS) showed that baby boomers were significantly more likely than Millennials to be victims of identity theft and that older people and minorities experience more severe economic and psychological consequences. This study examines how socioeconomic status, demographic characteristics, and incident-specific factors relate to how much money is stolen during identity theft, the likelihood of experiencing out-of-pocket costs, and emotional distress among identity theft victims age 65 and older. Using combined data from the 2014 and 2016 NCVS-ITS, this study examines the correlates of financial and psychological consequences of identity theft among 2,307 victims age 65 and older. Older Black victims are more likely to have greater amounts of money stolen and are more likely feel distressed than older non-Latino white identity theft victims. The most disadvantaged older adults living at or below the federal poverty level are nearly five times as likely to suffer out-of-pocket costs. The length of time information is misused and the hours spent resolving identity theft are significantly associated with emotional distress. More than one-third of older victims experience moderate to severe emotional distress following identity theft, and those who can least afford it suffer out-of-pocket costs. Greater advocacy and psychological support are needed to help older adults recover, in addition to tools to protect their personal information from misuse.

